# Characterizing the Processes for Navigating Internet Health Information Using Real-Time Observations: A Mixed-Methods Approach

**DOI:** 10.2196/jmir.3945

**Published:** 2015-07-20

**Authors:** Susan L Perez, Debora A Paterniti, Machelle Wilson, Robert A Bell, Man Shan Chan, Chloe C Villareal, Hien Huy Nguyen, Richard L Kravitz

**Affiliations:** ^1^ Betty Irene Moore School of Nursing University of California, Davis Sacramento, CA United States; ^2^ Department of Kinesiology and Health Science California State University, Sacramento Sacramento, CA United States; ^3^ Department of Internal Medicine University of California, Davis Sacramento, CA United States; ^4^ Department of Sociology University of California, Davis Davis, CA United States; ^5^ Department of Public Health Sciences, Division of Biostatistics University of California, Davis Sacramento, CA United States; ^6^ Department of Communication University of California, Davis Davis, CA United States; ^7^ Department of Public Health Sciences University of California, Davis Davis, CA United States; ^8^ Department of Infectious Disease University of California, Davis Sacramento, CA United States

**Keywords:** dual processing, information seeking, Internet search, health information

## Abstract

**Background:**

Little is known about the processes people use to find health-related information on the Internet or the individual characteristics that shape selection of information-seeking approaches.

**Objective:**

Our aim was to describe the processes by which users navigate the Internet for information about a hypothetical acute illness and to identify individual characteristics predictive of their information-seeking strategies.

**Methods:**

Study participants were recruited from public settings and agencies. Interested individuals were screened for eligibility using an online questionnaire. Participants listened to one of two clinical scenarios—consistent with influenza or bacterial meningitis—and then conducted an Internet search. Screen-capture video software captured Internet search mouse clicks and keystrokes. Each step of the search was coded as hypothesis testing (etiology), evidence gathering (symptoms), or action/treatment seeking (behavior). The coded steps were used to form a step-by-step pattern of each participant’s information-seeking process. A total of 78 Internet health information seekers ranging from 21-35 years of age and who experienced barriers to accessing health care services participated.

**Results:**

We identified 27 unique patterns of information seeking, which were grouped into four overarching classifications based on the number of steps taken during the search, whether a pattern consisted of developing a hypothesis and exploring symptoms before ending the search or searching an action/treatment, and whether a pattern ended with action/treatment seeking. Applying dual-processing theory, we categorized the four overarching pattern classifications as either System 1 (41%, 32/78), unconscious, rapid, automatic, and high capacity processing; or System 2 (59%, 46/78), conscious, slow, and deliberative processing. Using multivariate regression, we found that System 2 processing was associated with higher education and younger age.

**Conclusions:**

We identified and classified two approaches to processing Internet health information. System 2 processing, a methodical approach, most resembles the strategies for information processing that have been found in other studies to be associated with higher-quality decisions. We conclude that the quality of Internet health-information seeking could be improved through consumer education on methodical Internet navigation strategies and the incorporation of decision aids into health information websites.

## Introduction

The Internet has developed into a poorly organized information space of varying quality [1]. Its rapid growth has posed a serious problem due to people’s limited cognitive abilities to process the masses of information encountered during a typical Internet search [2]. Attempts have been made to understand the ways in which health information seekers cope with the unruly structure of the Internet. For example, investigators have examined the general intuitive strategies information seekers use to process complex online information [3-5].

In addition, several studies have used observational and survey methods to better understand how people undertake Internet health information searches in response to specific health-related questions and situations [3,6,7]. Findings from these studies suggest that health information search processes vary depending on current health circumstances and previous health experiences. In addition to situational factors, such as topic familiarity and complexity, there is evidence of variation in search strategies based on individual characteristics, such as gender, insurance status, education, and age [8]. If search patterns vary systematically by demographic and personal characteristics, it may ultimately be feasible to create targeted content and delivery systems that match up with group-level needs and preferences [9].

Our study focuses on consumers’ use of the Internet to interpret symptoms and reach a preliminary diagnosis. Such research is warranted by the fact that 35% of US adults use the Internet for self-diagnosis [7]. Specifically, we investigate the Internet health information search processes used to make health-related decisions amid the challenges that come with Internet navigation and the literacy levels required to decipher medical information [10].

Three main paradigms in psychology of judgment and decision making may inform how people seek information in response to acute, troubling symptoms [11]: (1) heuristics and biases research (also known as dual-processing theory) that focuses on an individual’s judgment of probability [12], (2) the study of decision making under risk [13], and (3) social judgment theory (as applied in the lens model) [14]. This investigation is grounded in dual-processing theory [15,16] because this theory emphasizes judgment under uncertainty [12] such as what one may encounter when seeking information to inform a response to troubling medical symptoms.

Dual-processing theory posits that two distinct cognitive systems (System 1 and System 2) are invoked during human decision making [12,17]. System 1 processing triggers the use of biases and heuristics, while System 2 processing is a methodical evaluation of the information presented. The use of System 2 for information processing may reduce the impact of the intuitive biases in the automated processes, which makes for sounder decision making [18]. Laymen and experts alike are prone to invoke heuristic biases characteristic of System 1 processing when pressed to think intuitively [19]. In order to reduce the biases imposed by System 1 thinking in order to arrive at a high-quality health decision, it is important to find ways to enforce System 2 processing. Understanding how Internet searchers enact strategies calling upon System 1 and System 2 thinking could have important implications for the way in which information on health websites is organized and presented.

An understanding of the processes by which Internet users seek, attend to, and assimilate health information can help webpage developers anticipate user needs and guide their attention to materials that support higher quality decisions [1,4]—decisions that are consistent with evidence-based practices and the patient’s own values. It may be particularly useful to understand when and how Internet health information seekers adopt System 1 versus System 2 processing. For all but the simplest decisions, the use of System 2 decreases bias and is associated with better decisions [18].

Additionally, an understanding of whether, how, and to what extent Internet users invoke information processing strategies during actual Internet health information searches could inform the design of Internet health search engines and websites [20]. Consider Internet users who invoke System 1 thinking when researching topics that are not familiar to them or topics that are complex. The introduction of features designed to slow down information-seeking processes, such as graphics, animations, sidebars, or quizzes, could prompt a shift towards System 2 thinking, leading to more deliberate, high-quality processing of information.

Previous studies have yet to characterize the processes by which individuals navigate Internet health information when making a health decision. We thus designed an observational study to explore strategies people use in seeking health-related information on the Internet and to understand the factors that predict their approach to finding and processing information. To standardize the stimuli provided to study participants, we used two clinical vignettes representing acute illnesses of contrasting clinical severity. There were no other constraints placed upon the participants as they investigated these two illnesses. In so doing, we aimed to describe Internet search processes and to identify demographic and personal characteristics associated with use of System 1 and System 2 cognitive processing.

## Methods

### Ethical Approval

The Institutional Review Board at the University of California, Davis, approved all study procedures.

### Recruitment of Participants

This investigation was designed as an exploratory, mixed-methods study focused on the Internet search patterns of young adults with limited access to a regular health care provider and services. Participant recruitment and data collection methods were adopted from previous studies [3,6]. We studied this age group because this demographic is more likely to experience barriers to health care continuity due to transitions in life and career [21], which may persist even with the implementation of improved coverage provisions such as the Affordable Care Act [22] and use of the Internet for health information [7,23]. The goal was to recruit at least 75 participants over a 6-month period. Participants were eligible if they were 21-35 years of age, had searched the Internet for health information within the past 12 months, and reported at least one of four barriers to accessing health care services (diminished ability to pay for services, no established relationship with a trusted primary care provider, an inability to get an appointment in a timely manner, or limited transportation options) [24].

Participants were recruited and data collected between March and August 2013. Potential participants were identified through door-to-door canvassing in a low-income housing community; canvassing at community fairs, community colleges, and local government offices that offer public services; sending emails through a University listserv targeting minority students; and posting flyers within the geographical region of Yolo County, California, at local coffee shops, public libraries, student family housing complexes, and community colleges. Individuals who expressed interest in participating were screened for eligibility using an online questionnaire (see Multimedia Appendix 1).

**Figure 1:**
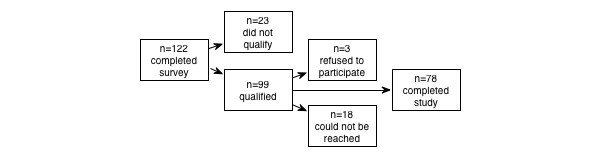
A breakdown of the number of individuals who completed the screening survey, qualified as eligible, agreed to participate, and completed the study.

In total, 78 of the 122 people screened were eligible (see Figure 1). Those who qualified and agreed to participate were contacted to schedule an interview at one of four public libraries.

### Study Procedures

Data were collected individually from each participant. Upon arrival at the study site, the participant was accompanied by the lead author to a quiet room or cubicle where they were provided written informed consent, participated in a brief demographic questionnaire, assessed for health status using the Short-Form (SF)-36 Health Survey [25], and instructed on study procedures and oriented to a laptop computer. For practice, participants were first asked to participate in a “mock search” focused on purchasing a box of chocolates. While conducting the mock search, subjects were asked to describe the actions they were taking, the content they were reading, and the qualities of the webpages that drew their attention. This instruction also oriented the participant to the operation of the laptop computer used for this study. Following this training exercise, the participant was randomly assigned to one of two clinical symptom scenarios of varying severity involving (1) fever, mild headache, dry cough, and myalgia, suggestive of influenza, and (2) fever, severe headache, and stiff neck, suggestive of meningitis. As a prompt to the clinical symptoms scenario, each participant was instructed to “Imagine you are experiencing this situation or think about a time when you had experienced this situation”. Unless the participant inquired, participants were not informed that the symptoms were suggestive of influenza or meningitis. See Multimedia Appendix 2 for an example of an Internet health information search.

The symptom scenarios were developed based on Centers for Disease Control and Prevention guidelines with input from clinical co-authors (RLK, HN). Both symptom scenarios were pilot tested for comprehensibility in a small sample of young adults (n=8). A total of 42 of the 78 participants were randomly assigned to the influenza scenario and 36 to the meningitis scenario. The participant was then instructed to “search the Internet, as though you were experiencing this situation” and trained to “think out loud” while doing so. The participant had a choice of Web browser that included Firefox, Internet Explorer, or Google Chrome. All Web browsers opened to a blank page. Participants’ Internet searches and think-out-loud vocalizations were digitally recorded using screen capture video-recording software [26]. Browser search history and cookies were deleted after each data collection session. Upon completion, the participant received a payment of US $20.

### Data Preparation and Coding

The digital video recordings were transferred from the laptop computer used for data collection to a computer used for analysis and saved to electronic files. While reviewing the video recordings of the Internet search, team members developed a list of interactions. “Interaction” was defined as the entering of a search term, selection of a website, and selection of a link to a website. We used these data to create a chronological workflow of each participant’s Internet search interactions. Members of the research team (SLP, CCV, and MSC) developed specific codes to be applied to the interactions through a process of analytical induction. Team members met regularly to compare their coding of participant’s interactions. Disagreements were resolved through discussion until members of the team reached consensus.

### Coding of Internet Search Behaviors

Each interaction was classified as one of three search units (SU): (1) hypothesis testing, (2) evidence gathering, or (3) treatment/action seeking [27]. Hypothesis testing describes interactions relevant to testing a diagnostic hypothesis (ie, entering the search term “meningitis” or clicking on a hyperlink titled “Flu”). Evidence gathering describes interactions involving symptoms (eg, “achy, high temperature, sore muscles” or clicking on the link “cough, muscle pain / symptom search”). Treatment/action seeking describes interactions that address remedies, recommended actions, or alerts such as recommendations for seeking immediate care from a health care provider, looking for a cure, or searching for health care services (ie, entering the search term “flu remedies” or selecting the link “When to Seek Medical Care”).

**Figure 2:**
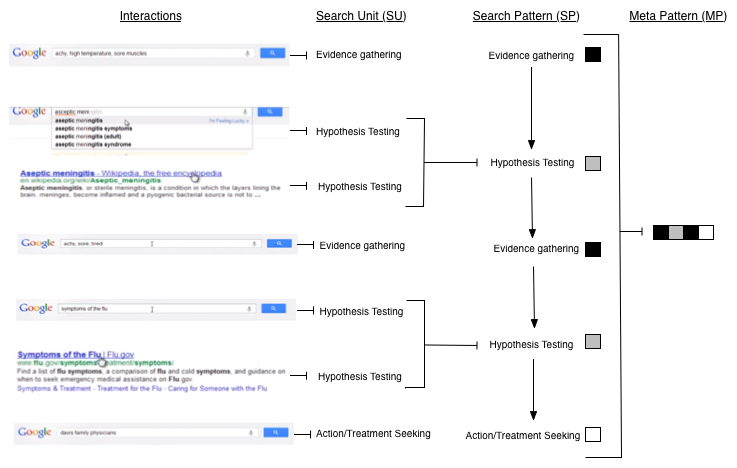
Example of coding process from interaction to meta-pattern.

Next, unbroken sequences of one, two, or more identical SUs were deemed search patterns (SPs). SPs are higher-order categories consisting of one or more SUs. For example, if a participant entered a query for “asceptic meni”, selected a link titled “aseptic meningitis”, and selected a link titled “Aseptic meningitis – Wikipedia the free encyclopedia”, these three consecutive hypothesis testing SUs would be merged to form one hypothesis testing SP as shown in the middle panel of Figure 2.

As the last step in the Internet search coding process, SPs for a given individual were ordered chronologically. The resulting sequences were displayed graphically and then categorized into a small set of overarching patterns called meta-patterns (MPs). Figure 2 provides an example of the procedure for converting Internet interactions into a MP. This example forms the following meta-pattern:

evidence gathering → hypothesis testing → evidence gathering → hypothesis testing → action/treatment seeking

The MPs were organized into four overarching pattern classifications using a hierarchical system for classification based on number of SPs (≥1 and <1), inclusion of a hypothesis testing and/or evidence gathering SP pattern combination (in no specific order), and inclusion of action/treatment seeking before termination of the search. Finally, the four overarching pattern classifications were examined in light of the dual-processing theory framework, allowing each Internet search to be categorized as predominantly System 1 or System 2. The patterns classified as including a hypothesis testing and evidence gathering SP combination (in no specific order) were categorized as System 2. All other pattern classifications were categorized as System 1.

### Statistical Analysis

We identified demographic and personal characteristic differences between respondents using System 1 processing and those using System 2. A *t* test was used to make this two-group comparison for age in years, as well as SF-36 scales assessing physical functioning, role-physical, bodily pain, general health, vitality, social functioning, role-emotional, and mental health [25]. The chi-square test was used for the categorical variables of race, gender, treatment, recruitment site, and education level. Respondent characteristics with a *P* value ≤.1 or less were included in a multivariate logistic regression model. In this analysis, dominant search strategy served as the dependent variable, with System 1 serving as the reference group. We fit a logistic regression model using a backward selection procedure to test for effects of the SF-36 scale physical functioning, site, gender, race, and education. A *P* value of .05 was considered significant. All statistical analyses were performed using SAS(r) software version 9.3.

## Results

### Sample Characteristics

A summary of participant demographic characteristics is presented in Table 1. The sample was young, with a mean age of 25 (SD 4.38), predominantly female, nonwhite with a strong Hispanic representation, not college educated, and health-insured. When comparing the average values of study participants’ demographic characteristics with residents of Yolo County [28,29] as a whole, all the variables, except education, were significantly different (*P*>.05). In order to conserve power, the racial groups of Asian (4/78, 5%), American Indian or Alaska Native (2/78, 3%), Black/African American (3/78, 4%), mixed race (4/78, 5%), other (29/78, 37%), and those who declined to state (13/78, 17%) were categorized as “Other”.

**Table 1:** Summary of participant demographic characteristics.

Category		Study participants (N=78) n (%)	All Yolo County residents^a^ (N=200,849) %
Age in years, mean		25	30^c^
**Gender**			
	Male	23 (29)	49
	Female	55 (71)	51
**Race**			
	White	23 (29)	76
	Other	55 (71)	24
**Education**			
	No bachelor’s degree	52 (66)	62
	Bachelor’s degree or higher	26 (35)	38
**Insurance status**			
	Uninsured	18 (23)	20^b^
	Public insurance	11 (14)	19^b^
	Other insurance	49 (63)	68^b^
Total			

^a^2010 US Census [28].

^b^Among individuals under 65 years old; 2005 California Health Interview Survey [29].

^c^City-data.com.

### Internet Search Patterns

**Figure 3:**
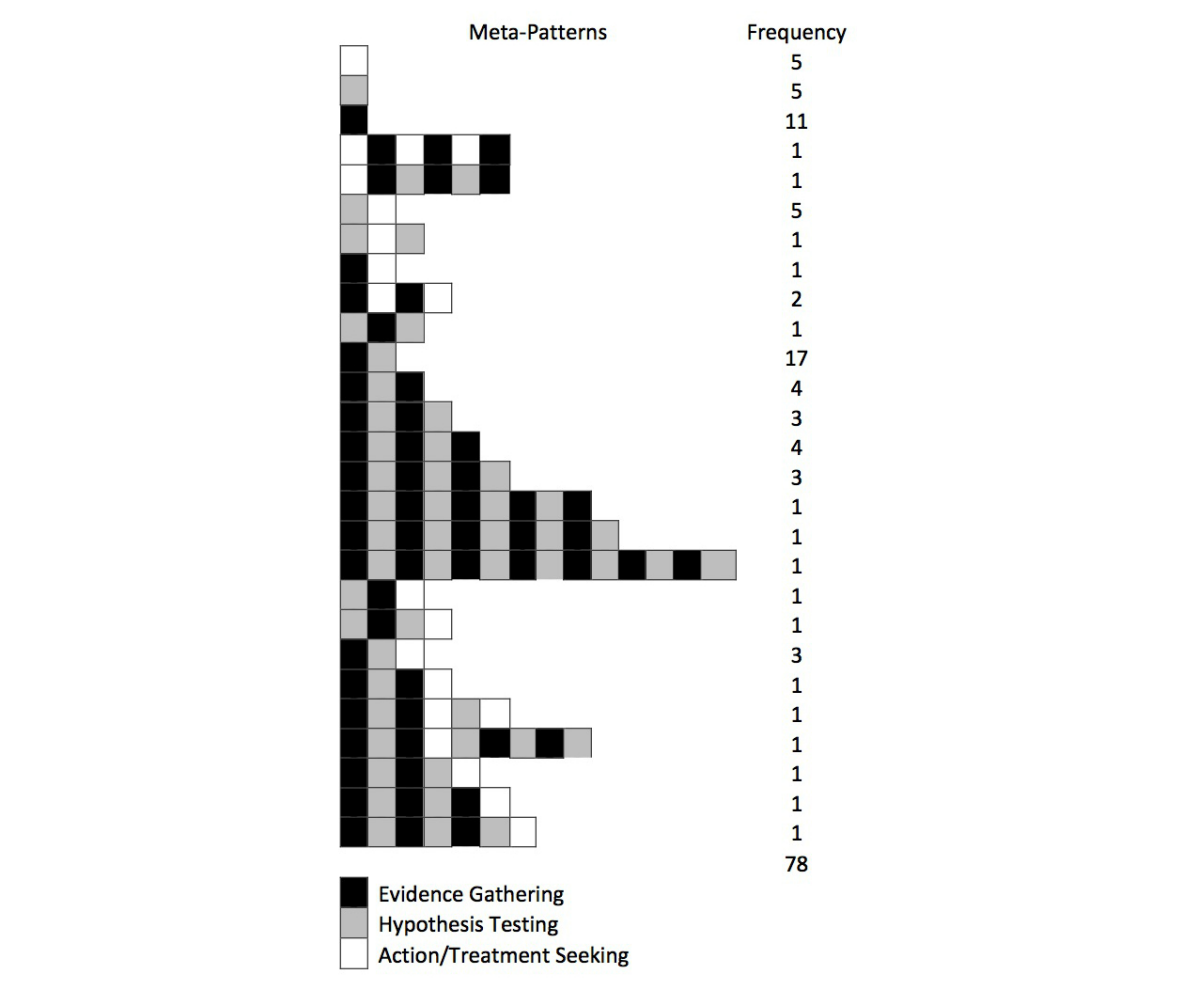
Frequency of each meta-pattern observed.

The duration of Internet searching ranged from 0.92 minutes to 14.27 minutes with an average of 5.13 minutes. There was a moderate positive correlation between the number of interactions (eg, mouse clicks and entering search terms) and duration of Internet searching: *r*=.38, *P*<.001. There was wide variation in the number and ordering of search patterns (SPs). We identified 27 unique pattern variations of MPs as shown in Figure 3.

**Figure 4:**
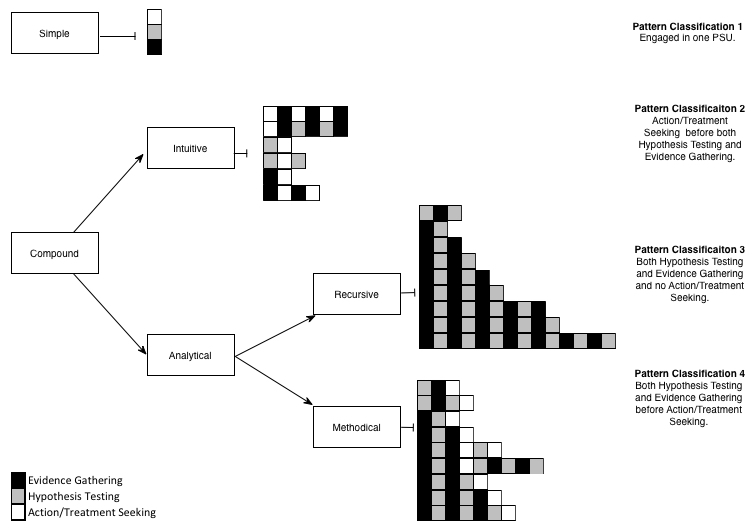
Classification of search strategies.

Working by consensus of the co-authors, we identified four overarching pattern classifications as depicted in Figure 4. These four overarching pattern classifications were created by first grouping search patterns based on number of steps taken during the search. A *simple* search was one involving a single step (21/78, 27%). A compound search was any search involving two or more steps (57/78, 73%).

Thereafter, each compound pattern was placed into one of two subgroups, labeled *intuitive* and *analytical*. An intuitive search was any search that involved action/treatment seeking before hypothesis testing and evidence gathering were carried out. An analytical search was one that began with hypothesis testing and evidence gathering. An analytical search that did not lead to action/treatment seeking was further classified as *analytical-recursive*. An analytical search that lead to action/treatment seeking was classified as *analytical-methodical*. Of the 57 compound searches, 11 (19%) were intuitive, 31 (54%) were analytical-recursive, and 15 (26%) were analytical-methodical.

The four overarching pattern classifications we derived inductively fit neatly within the dual-processing theory for information processing. Pattern Classifications 1 and 2 were characterized as *System 1 processing* because these search patterns involved rapid progression to action/treatment or terminated after limited Internet searching, implying reliance on heuristic cues or ready satisfaction with Internet health information. Pattern Classifications 3 and 4 were characterized as *System 2 processing* (systematic) because these patterns involved a systematic approach to searching for information about specific diagnoses and symptoms prior to a search for actions/treatments or search termination. Of the 78 respondents, 32 (41%) engaged in System 1 processing and 46 (59%) relied on System 2 processing.

### Predictors of Systematic Searching

A backward stepwise binary logistic regression model was constructed to predict a systematic approach to searching Internet health information (compared with System 1 processing) using age, clinical symptom scenario (influenza vs meningitis), male sex, white race, college education, and recruitment site not located within a university town as predictors (independent variables). The resulting model (Table 2) revealed a strong association between choice of systematic processing with education and age. Systematic processing was not significantly associated with symptom scenario, gender, race, or insurance status. For every 1-year increase in age, the odds of systematic processing decreased by 13.3% (OR 0.87, 95% CI 0.77-0.98, *P*=.02). There was also a significant negative effect of education on central route processing (OR 0.30, 95% CI 0.09-0.94, *P*=.04). Less educated participants (those without a bachelor’s degree) were less likely to use systematic processing. There also were significant effects of recruitment site.

**Table 2:** Binary logistic regression analysis of the relationship of demographic characteristics with reliance on systematic search strategies (N=78).

Variable	OR	95% CI	*P*
Age	0.87	0.77-0.98	.02
Education (no bachelor’s degree)	0.30	0.09-0.94	.04
Recruitment site	3.75	1.29-10.92	.02

## Discussion

### Principal Findings

In this study, we directly observed young adults as they searched for information on two hypothetical clinical scenarios varying in severity, influenza, and meningitis. The four overarching pattern classifications were categorized into two information-processing strategies as postulated by dual-process theory. While the results demonstrate a modest preference for behaviors associated with System 2 thinking, a substantial proportion of study participants relied on simple or “intuitive” approaches associated with System 1. In terms of predictors, we found that younger participants and those with more education were more likely embrace a System 2 approach.

Previous research on Internet health information seeking has focused on Internet information accuracy [10,30-36], completeness [30,32,36,37], and readability by the lay public [10,38]. Other investigators have focused on user information preferences and needs [3], demographics of individuals with specific information preferences [7], and user accounts of preferences for information format [3]. These studies, evaluating Internet health information and user characteristics, suggest that Internet health information is often inaccurate, incomplete, and difficult to comprehend by lay audiences, yet individuals are still turning to the Internet for health decision making [7].

Little can be done to address the inconsistencies of the millions of Internet health information websites [39], but we can find ways to guide Internet health information seekers toward information processing strategies that may be more likely to lead to accurate decision making. Prior research concerning the design of information systems indicates that to support accurate decision making, systems should be process-oriented rather than information-oriented because information seeking and decision making involve a series of encounters over time rather than a single information encounter [40,41]. The distinction between automatic (System 1) and controlled (System 2) processing explained the roles of motivation and cognitive ability in the decision-making processes of the young adults studies [20].

The decision-making process most conducive to a high-quality decision involves systematically gathering all available information about a situation, weighing every feasible option, and integrating the available data to make the decision most likely to produce desired outcomes [42]. When there is no single “best” action, a high-quality decision balances the subjective values of the consumer’s assessment of benefits versus harms [43]. Of the two observed approaches to Internet health information seeking, the behaviors associated with System 2 may be most conducive to a high-quality decision because System 2 processors methodically develop a hypothesis (eg, a provisional conjecture established from information gathered during the Internet search or previously held knowledge) and gather information (eg, gathering information to confirm or develop a hypothesis) before taking action/treating or terminating their search.

System 1 health information seekers are more likely to reach decisions based on simplifying heuristic rules and to terminate their search once they have found an acceptable solution, not necessarily the best. The System 1 approach is often effective because it economizes on time and usually leads to sensible decisions [42]. However, departures from the ideal strategy of information seeking may lead to mistakes, such as errors in reasoning that arise from misinformation, denial, overconfidence, distrust, or confusion [42].

### Limitations

This study has limitations. First, generalizability is limited by a small sample size of young adults recruited by convenience sampling from a limited geographical region in central California. Second, the participant characteristics of gender, race, and insurance status did not fully reflect the demographics of the region from which participants were recruited. Third, participants may have had more or less familiarity with the symptom scenarios, which could bias their search process as a result of previous clinical experiences. Fourth, the unnatural and forced environment of our experiment may have influenced how subjects searched. Fifth, we likely omitted variables or factors that would potentially affect Internet search patterns. Finally, given their exploratory nature, these findings need to be validated with fresh samples.

Due to the unregulated nature of the Web itself, Internet health information seekers are susceptible to a wayward and distracted process of information gathering. Methodical Internet searching to guide health information seekers toward high-quality decisions should be approached in two ways: (1) through consumer education on methodical Internet navigation strategies and (2) through the incorporation of decision aids into health information websites.

Consumer education on a methodical approach to decision making when using the Internet would include guidance on the process of first defining the decision, then gathering information about potential decision outcomes, and ensuring that the final decision is consistent with the consumer’s values [43]. The incorporation of decision aids into Internet health information websites should consider (1) defining the decision (health information resources should provide information about all options), (2) providing information about potential decision outcomes (health information resources should present probabilities, balance the presentation of options, and base information on up-to-date scientific evidence), and (3) supporting decision making that is consistent with consumers’ values (health information resources should clarify and express values, use patient stories, and guide the deliberation process) [44].

Further research is needed to confirm the information-seeking processes most conducive to supporting high-quality decisions leading to the best possible outcomes. Professional health care providers can do little to control the type of health information encountered on the Internet, but they can help steer their patients towards Internet resources that encourage deliberative thinking and thus better decision making.

## Appendix

Multimedia Appendix 1 Online questionnaire used to screen for eligibility to participate.

Multimedia Appendix 2 Example of an Internet health information search, with annotations, in response to a vignette of symptoms mimicking meningitis.

## Abbreviations

MP: meta-patterns
SF-36: Short-Form-36 Health Survey
SP: search patterns
SU: search units

## References

[1] Hölscher C, Strube G (2000). Web search behavior of Internet experts and newbies. Computer Networks.

[2] Goldsmith J (2000). How will the Internet change our health system?. Health Affairs.

[3] Eysenbach G, Köhler C (2002). How do consumers search for and appraise health information on the world wide web? Qualitative study using focus groups, usability tests, and in-depth interviews. BMJ.

[4] Horvitz E, Kadie C, Paek T, Hovel D (2003). Models of attention in computing and communication. Commun. ACM.

[5] Gil M, Serral E, Valderas P, Pelechano V (2013). Designing for user attention: A method for supporting unobtrusive routine tasks. Science of Computer Programming.

[6] van Deursen Alexander J A M, van Dijk Jan A G M (2011). Internet skills performance tests: are people ready for eHealth?. J Med Internet Res.

[7] Fox Susannah, Duggan Maeve (2013). Pew Internet and American Life Project.

[8] Anker AE, Reinhart AM, Feeley TH (2011). Health information seeking: a review of measures and methods. Patient Educ Couns.

[9] Suggs LS, McIntyre C (2009). Are we there yet? An examination of online tailored health communication. Health Educ Behav.

[10] Berland G, Elliott M, Morales L, Algazy J, Kravitz R, Broder M, Kanouse D, Muñoz J, Puyol J, Lara M, Watkins K, Yang H, McGlynn E (2001). Health Information on the Internet. JAMA.

[11] Evans Jonathan St B T (2008). Dual-processing accounts of reasoning, judgment, and social cognition. Annu Rev Psychol.

[12] Holyoak K, Morrison R, Frederick S, Holyoah K, Morrison R (2005). A Model of Heuristic Judgment. The Cambridge handbook of thinking and reasoning.

[13] Harvey N, Koehler DJ, Zhang J, Gonzalez R, Koehler D (2004). Decision under risk. Blackwell handbook of judgment and decision making.

[14] Johnson W, Doherty M (1983). Social judgment theory and academic advisement. Journal of Counseling Psychology.

[15] Chaiken S (1980). Heuristic versus systematic information processing and the use of source versus message cues in persuasion. Journal of Personality and Social Psychology.

[16] Petty Richard, Cacioppo John T (1986). Communication and Persuasion: Central and Peripheral Routes to Attitude Change.

[17] World Health Organization (1982). Control of Health Care Costs in Social Security Systems. Report on a Workshop.

[18] Kahneman D, Lovallo Dan, Sibony Olivier (2011). Before you make that big decision. Harv Bus Rev.

[19] Tversky A, Kahneman D (1974). Judgment under Uncertainty: Heuristics and Biases. Science.

[20] Metzger M (2007). Making sense of credibility on the Web: Models for evaluating online information and recommendations for future research. J Am Soc Inf Sci.

[21] Schwartz Karen, Sommers Benjamin D (2012). ASPE Research Brief.

[22] Shartzer Adele, Long Sharon K, Benatar Sarah (2015). Health Reform Monitoring Survey.

[23] Fronstin Paul (2013). California Healthcare Foundation.

[24] (2015). Health People 2020.

[25] Ware JE, Gandek B (1998). Overview of the SF-36 Health Survey and the International Quality of Life Assessment (IQOLA) Project. J Clin Epidemiol.

[26] (2015). Camtasia Studio 8.

[27] Kassirer J, Wong JK, Kopelman RE (2009). Learning Clinical Reasoning.

[28] (2010). State and County QuickFacts.

[29] (2005). California Health Interview Survey.

[30] Impicciatore P, Pandolfini C, Casella N, Bonati M (1997). Reliability of health information for the public on the World Wide Web: systematic survey of advice on managing fever in children at home. BMJ.

[31] Jejurikar SS, Rovak JM, Kuzon WM, Chung KC, Kotsis SV, Cederna PS (2002). Evaluation of plastic surgery information on the Internet. Ann Plast Surg.

[32] Kulasegarah J (2007). The quality of information on three common ENT procedures on the Internet. Ir J Med Sci.

[33] Kunst H, Groot D, Latthe PM, Latthe M, Khan KS (2002). Accuracy of information on apparently credible websites: survey of five common health topics. BMJ.

[34] Quinn E, Corrigan M A, McHugh S M, Murphy D, O'Mullane J, Hill A D K, Redmond H P (2012). Breast cancer information on the internet: analysis of accessibility and accuracy. Breast.

[35] Scullard P, Peacock C, Davies P (2010). Googling children's health: reliability of medical advice on the internet. Arch Dis Child.

[36] Zun LS, Downey L, Brown S (2011). Completeness and accuracy of emergency medical information on the web: update 2008. West J Emerg Med.

[37] Frické M, Fallis D, Jones M, Luszko GM (2005). Consumer health information on the Internet about carpal tunnel syndrome: indicators of accuracy. Am J Med.

[38] Hendrick P, Ahmed Osman H, Bankier Shane S, Chan Tze Jieh, Crawford Sarah A, Ryder Catherine R, Welsh Lisa J, Schneiders Anthony G (2012). Acute low back pain information online: an evaluation of quality, content accuracy and readability of related websites. Man Ther.

[39] Kontos E, Bennett G G, Viswanath K (2007). Barriers and facilitators to home computer and internet use among urban novice computer users of low socioeconomic position. J Med Internet Res.

[40] Soergel D (1985). Organizing information: principles of data base and retrieval systems.

[41] Kuhlthau C (1991). Inside the Search Process: Information Seeking from the User Perspective. Journal of the American Society for Information Science.

[42] Redelmeier DA, Rozin P, Kahneman D (1993). Understanding patients' decisions. Cognitive and emotional perspectives. JAMA.

[43] Stacey Dawn, Legare France, Nananda F Col, Bennett Carol L (2014). Decision aids for people facing health treatment or screening decisions. Cochrane Database Syst Rev.

[44] Elwyn Glyn, O'Connor Annette, Stacey Dawn, Volk Robert, Edwards Adrian, Coulter Angela, Thomson Richard, Barratt Alexandra, Barry Michael, Bernstein Steven, Butow Phyllis, Clarke Aileen, Entwistle Vikki, Feldman-Stewart Deb, Holmes-Rovner Margaret, Llewellyn-Thomas Hilary, Moumjid Nora, Mulley Al, Ruland Cornelia, Sepucha Karen, Sykes Alan, Whelan Tim, International Patient Decision Aids Standards (IPDAS) Collaboration (2006). Developing a quality criteria framework for patient decision aids: online international Delphi consensus process. BMJ.

