# Supplementary material for: Characterizing the Processes for Navigating Internet Health Information Using Real-Time Observations: A Mixed-Methods Approach
Source: J Med Internet Res. 2015 Jul 20;17(7):e173. doi: 10.2196/jmir.3945 (PMC4527005; doi:10.2196/jmir.3945)
Supplement: Multimedia Appendix 1 [file jmir_v17i7e173_app1.pdf]

Internet Health Information Seeking

We are interested in the ways people are using Internet health information to make health decisions. This is a survey to see if you are eligible to participate in our study.

Thank you!

Please complete the survey below.

Are you between the ages of 18 and 35 years old?
- ☐ Yes
- ☐ No

Internet for Health Information

In the past 12 months, have you used the Internet to look for health or medical information for yourself?
- ☐ Yes
- ☐ No

Health Care Services Utilization and Access

Do you have a personal doctor or medical provider who is your main provider?
- ☐ Yes
- ☐ No
- ☐ Refused
- ☐ Don't know

During the past 12 months, did you phone or e-mail the doctor's office with a medical question?
- ☐ Yes
- ☐ No
- ☐ Refused
- ☐ Don't know

How often did you get an answer as soon as you needed it?
- ☐ Never
- ☐ Sometimes
- ☐ Usually
- ☐ Always
- ☐ Refused
- ☐ Don't know

During the past 12 months, did you delay or not get any other medical care you felt you needed-- such as seeing a doctor, a specialist, or other health professional?
- ☐ Yes
- ☐ No
- ☐ Refused
- ☐ Don't know

Was cost or lack of insurance a reason why you delayed or did you get the care you felt you needed?
- ☐ Yes
- ☐ No
- ☐ Refused
- ☐ Don't know

In the past 12 months, how often did you feel you could rely on your doctors, nurses, or other health care professionals to take care of your health care needs?
- ☐ Always
- ☐ Usually
- ☐ Sometimes
- ☐ Never

How would you normally travel to see a health care provider?
- ☐ Drive myself
- ☐ Have a friend/family member drive me
- ☐ Take public transportation
